# Phenolic-rich extruded BRS 305 sorghum-based beverage improves fecal and blood metabolites, oxidative balance and cardiometabolic markers in adults with excess body weight: a single-blind, randomized, placebo-controlled study

**DOI:** 10.1007/s00394-026-04019-2

**Published:** 2026-06-19

**Authors:** Lucimar Aguiar da Silva, Vinícius Parzanini Brilhante de São José, Álvaro Luiz Miranda Piermatei, Larissa Arruda Rodrigues, Pietra Vidal Cardoso do Prado, Renata Celi Lopes Toledo, Carlos Wanderlei Piler de Carvalho, Valéria Aparecida Vieira Queiroz, Bárbara Pereira da Silva, Joseph Francis Pierre, Hércia Stampini Duarte Martino

**Affiliations:** 1 Department of Nutrition and Health, Federal University of Viçosa, Viçosa, Brazil; 2 Embrapa Agroindústria de Alimentos, Rio de Janeiro, Rio de Janeiro, Brazil; 3 Embrapa Milho e Sorgo, Minas Gerais Sete Lagoas, Brazil; 4 Department of Nutritional Sciences, University of Wisconsin-Madison, Madison, USA

**Keywords:** Plasma phenolic profile, Total antioxidant capacity, Gastrointestinal health, Acetic acid, *Sorghum bicolor* (L.) Moench

## Abstract

**Purpose:**

To evaluate the impact of a phenolic-rich, extruded BRS305 sorghum-based beverage on fecal and blood metabolic, oxidative balance, and cardiometabolic parameters in adults with excess body weight.

**Methods:**

In a single-blind, randomized, placebo-controlled study, 51 overweight adults of both sexes, were assigned to consume a sorghum beverage (SG, *n* = 25) or a control beverage (CG, *n* = 26), daily for 10 weeks, alongside a personalized hypocaloric diet (−500 kcal). The beverages phenolic profile was analyzed. Anthropometry, body composition, food intake, biochemicals, total plasma antioxidant capacity, oxidative stress, blood metabolite, fecal pH, short-chain fatty acids concentration, and Bristol stool scale were assessed at baseline and endpoint.

**Results:**

The sorghum beverage exhibited high diversity of phenolic compounds, including phenolic acids, flavonoids, and tannins, which reflected in higher plasma concentrations of *trans*-caffeic acid and naringenin in SG at the endpoint compared to the CG, along with an increase in total plasma antioxidant capacity. In the SG, superoxide dismutase and malondialdehyde decreased in intra-group, while catalase decreased at delta. In contrast, CG decreased nitric oxide levels and increased body fat percentage in intra-group. SG decreased insulin, triglycerides, HOMA-IR, and TyG index intra-group, maintaining body fat percentage; increased HDL-c and decreased Castelli I index at the endpoint; and decreased Castelli II index at delta. SG also decreased fecal pH and increased acetic acid content at endpoint and delta.

**Conclusion:**

These findings support the functional potential of extruded BRS305 sorghum-based beverage in improving fecal and blood metabolites, oxidative balance and cardiometabolic markers in adults with excess body weight.

**Graphical abstract:**

**Figure:**
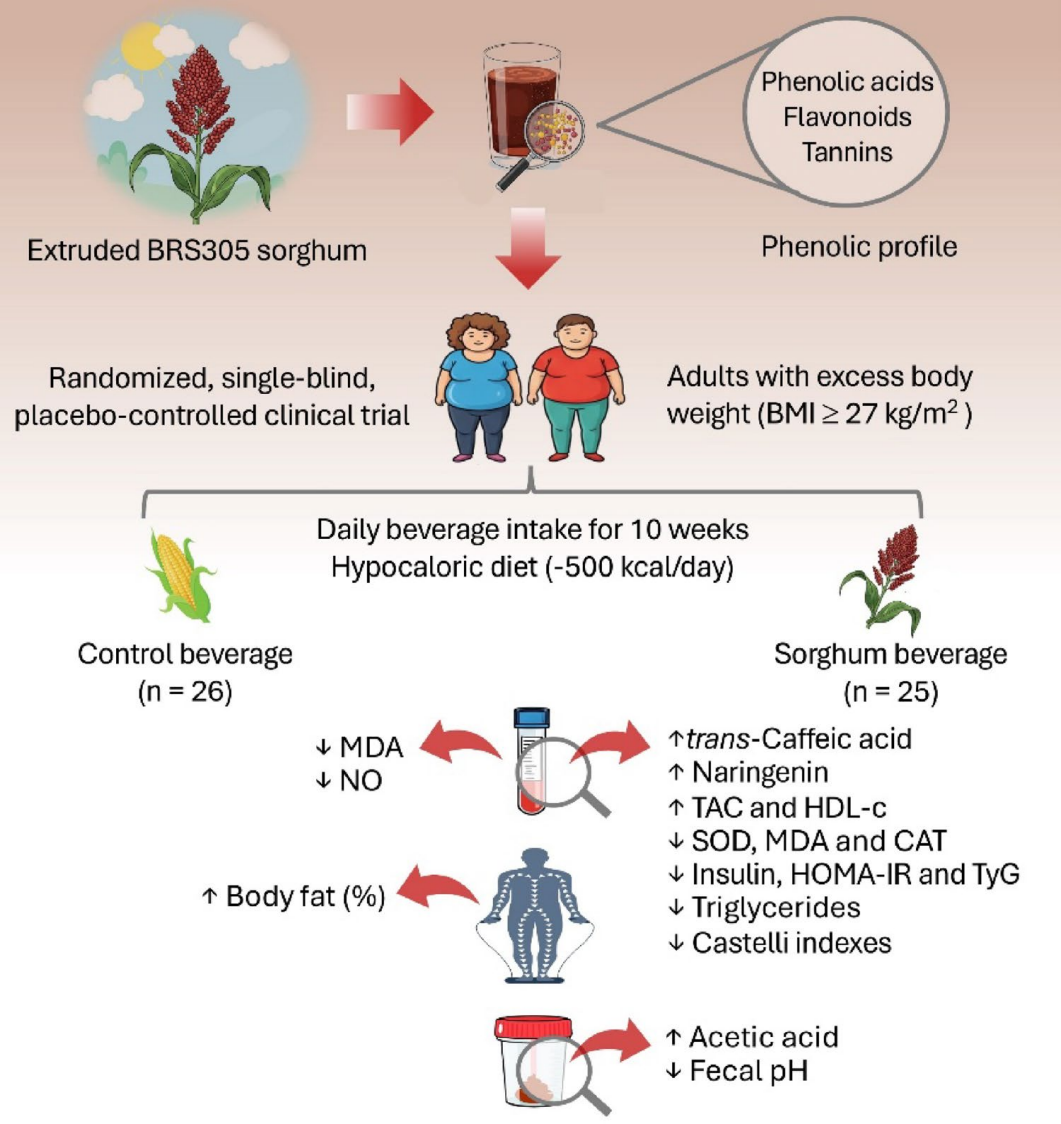

**Supplementary Information:**

The online version contains supplementary material available at 10.1007/s00394-026-04019-2.

## Introduction

 The global rise in overweight and obesity has reached alarming proportions, significantly contributing to the development of chronic diseases such as type 2 diabetes, and cardiovascular disorders [1]. Central to these conditions are metabolic dysfunction, and oxidative stress, all of which are strongly influenced by dietary patterns [2]. Given the multifactorial nature of obesity, there is an increasing interest in study how functional foods are capable of modulating multiple physiological pathways in the body [3].

Within this context, whole grains, particularly those rich in phenolic compounds and dietary fiber, have attracted attention for their ability to modulate body composition, enhance systemic antioxidant defenses, and improve intestinal health [4]. Among those, sorghum (*Sorghum bicolor* (L.) Moench) stands out as a gluten-free, climate-resilient cereal with a remarkable phytochemical profile. The BRS 305 genotype, produced in Brazil, present high concentrations of resistant starch, and bioactive phenolic compounds such as protocatechuic acid, *trans*-caffeic acid, naringenin, catechins, and procyanidins, compounds associated with antioxidant defense, and cardiometabolic markers [5–7].

Although promising effects of sorghum have been demonstrated in in vitro and animal studies [6, 8, 9], human clinical trials, particularly those involving the BRS 305 genotype, remain limited. Furthermore, the potential of sorghum-based beverages to impact multiple health-related outcomes, such as gut health, oxidative balance, and cardiometabolic markers in adults with excess body weight, has not been fully explored.

Beyond its inherent nutritional and phytochemical properties, the functional potential of sorghum can be enhanced through extrusion cooking, a thermomechanical process widely applied in the production of ready-to-eat foods [10]. This process alters the physical and chemical structure of grains, improving digestibility and functional properties [11]. Specifically, in sorghum, extrusion cooking has been shown to increase the bioacessibility and bioavailability of phenolic compounds, thereby potentially amplifying their health benefits [12]. It may also improve sensory characteristics, making extruded sorghum-based products more effective and acceptable for human consumption [13, 14]. Thus, incorporating extruded BRS 305 sorghum into a beverage format offers a promising strategy to deliver bioactive compounds in a more palatable and bioavailable form.

Anunciação et al. [15] found that extruded sorghum consumption decreased body fat percentage and increased the serum glutathione peroxidase concentration in overweight men [15]. In a pilot study from our research group, the extruded BRS305 sorghum beverages, with or without *Lacticaseibacillus paracasei*, demonstrated favorable nutritional value and improved body composition, lipid-related cardiovascular risk (Castelli I index), and intestinal health in overweight and obese adults [16]. Thus, this study aimed to evaluate the effects of a phenolic-rich extruded BRS 305 sorghum-based beverage on fecal and blood metabolites, oxidative balance, and cardiometabolic markers in adults with overweight and obese. It was hypothesized that the phenolic compounds present in the extruded BRS 305 sorghum beverage exert bioactive effects, improving fecal and blood metabolites, oxidative balance, and cardiometabolic markers.

## Materials and methods

### Development of sorghum and control beverages

For the sorghum beverage, whole grains of the BRS 305 genotype were produced and cleaned by *Embrapa Milho e Sorgo* (Sete Lagoas, Minas Gerais, Brazil) and sent to *Embrapa Agroindústria de Alimentos* (Rio de Janeiro, Rio de Janeiro, Brazil), where the extrusion cooking process was performed using a co-rotating twin-screw extruder, the Evolum HT25 (Clextral Inc., Firminy, France) and detailed by Da Silva et al. [16]. The resulting expanded or puffed extrudates were dried and then milled into fine flour used in the formulated beverage described as following.

The sorghum beverage was developed using extruded sorghum flour (11.54%), maltodextrin (11.54%), and soy milk (38.46%), and was flavored with pure juice in various flavors (apple, mango, guava, grape, and peach) (38.46%). The control beverage had an identical composition as the test beverages, except that the waxy maize starch (Growth Supplements→, Tijucas, Santa Catarina, Brazil) was used in place of extruded sorghum flour. The final beverage volume was approximately 250 mL for women and 340 mL for men, adapted to replace the total caloric intake of breakfast (15% of the daily calorie requirement, based on a 2,000-kilocalorie diet for women and 2500-kilocalorie diet for men). The beverages were prepared, lyophilized, and analyzed to determine their nutritional composition for labeling purposes. A phenolic extract was specifically prepared for total phenolic content determination by adding 0.5 g of lyophilized beverage sample to 5 mL of a methanol: water solution (60:40, v/v), followed by stirring for 16 h at room temperature. Total phenolic content was analyzed using the Folin–Ciocalteu method, and the result was expressed as milligrams of gallic acid equivalents [16] (Table 1).

Beverages were prepared and distributed to volunteers on a weekly basis, along with instructions to store them under refrigeration (4 °C), consumed one bottle daily at breakfast, and shook well immediately before consumption. At the end of each week, volunteers returned the empty bottles along with a daily consumption record.

**Table 1:** Nutritional composition of sorghum (a) and control (b) beverages

(a)				(b)			
Nutrition facts				Nutrition facts			
1 serving per container - Serving size: 250 mL				1 serving per container - Serving size: 250 mL			
	100 mL	250 mL	% DV*		100 mL	250 mL	% DV*
Calories (kcal)	112.08	280.20	14%	Calories (kcal)	115.39	288.48	14%
Total carbohydrate (g)	24.51	61.28	20%	Total carbohydrate (g)	25.84	64.60	22%
Dietary fiber (g)	0.80	2.00	8%	Dietary fiber (g)	0.00	0.00	0%
Total sugars (g)	23.71	59.28	20%	Total Sugars (g)	25.84	64.60	22%
Added sugars	0.00	0.00	0%	Added Sugars	0.00	0.00	0%
Protein (g)	3.59	8.98	18%	Protein (g)	2.49	6.23	12%
Total Fat (g)	0.32	0.80	1%	Total fat (g)	0.23	0.58	1%
Saturated Fat (g)	0.00	0.00	0%	Saturated fat (g)	0.00	0.00	0%
*Trans* Fat (g)	0.00	0.00	0%	*Trans* fat (g)	0.00	0.00	0%
Cholesterol (mg)	**	**	**	Cholesterol (mg)	**	**	**
Sodium (mg)	**	**	**	Sodium (mg)	**	**	**
Total phenolic compounds (mg GAE)	618.29	1600.28	**	Total phenolic compounds (mg GAE)	70.21	181.72	**

*The % Daily Value (DV) tells you how much a nutrient in a serving of food contributes to a daily diet. 2,000 kilocalories a day is used for general nutrition advice. ** Not analyzed. mg GAE: milligrams of gallic acid equivalents.

### Phenolic compound profile of sorghum and control beverages

After preparation, the sorghum and control beverages were freeze-dried (Labconco Corp., Missouri, USA) until complete dehydration, with an average processing time of 12 h.

For the extraction of phenolic compounds, 5 g of the freeze-dried beverage flours were extracted with 30 mL of methanol: water (80:20, v/v) by shaking at room temperature (25 °C) for 2 h. The extract was centrifuged at 2358 *g* at 20 °C for 10 min. The supernatant was decanted, and the residue was extracted once more under the same conditions. The two supernatants were combined and stored in a refrigerator below 5 °C prior to analysis.

UHPLC-Q-orbitrap HRMS analysis was conducted using a Vanquish UHPLC coupled to an Orbitrap Exploris 240 (Thermo Fisher Scientific, Waltham, MA, USA). The column used for LC separation was HSS T3 (100 ⋅ 2.1 mm, 1.8 μm, Waters, Santa Clara, CA, USA). The solvents used were (A) 0.1% formic acid in water and (B) 0.1% formic acid in methanol. The flow rate was 0.3 mL/min, and the injection volume was 5 µL. Thermo Scientific™ Xcalibur™ 4.2 software (Thermo Fisher Scientific, Waltham, MA, USA) was used for targeted compound data analysis. Compound Discover 3.3 software (Thermo Fisher Scientific, Waltham, MA, USA) was used for further data analysis.

This analysis identified 511 compounds (Supplementary Material 1). After excluding duplicates, non-phenolic compounds, and substances that are poorly known or rarely studied, 124 phenolic compounds were identified. Among these, 94 were exclusive to the extruded BRS 305 sorghum beverage, while 30 were present in both sorghum and the control beverages. The 12 main compounds identified are presented in Table 2, and the chromatograms of the sorghum and control beverages are presented in Supplementary Material 2. The sorghum beverage demonstrated a higher diversity of phenolic compounds compared to the control beverage. Among the phenolic compounds presents in both beverages, the sorghum beverage contained higher abundance of protocatechuic acid, *trans*-caffeic acid and naringenin, based on chromatographic peak areas. The selected phenolic compounds were targeted for plasma quantification in study participants.

**Table 2:** Identification of phenolic compounds in sorghum and control beverages obtained by UHPLC

Phenolic compound	Molecular formula	RT (min)	m/z	Control beverage	Sorghum beverage	Higher in sorghum beverage ^d^
Phenolic acids						
Protocatechuic acid ^a^	C7 H6 O4	7.63	153.02	+	+	Y
*trans*-Caffeic acid ^a^	C9 H8 O4	20.29	179.03	+	+	Y
*trans*-Ferulic acid ^a^	C10 H10 O4	29.15	193.05	+	+	N
5-Caffeoylquinic acid ^a^	C16 H18 O9	11.37	353.09	−	+	N/A
*Flavonoids*						
Naringenin ^a^	C15 H12 O5	36.02	271.06	+	+	Y
Quercetin ^a^	C15 H10 O7	36.03	301.04	+	+	N
Daidzein ^a^	C15 H10 O4	35.83	253.05	+	+	N
Eriodyctiol ^a^	C15 H12 O6	35.49	287.06	+	+	N
Taxifolin ^b^	C15 H12 O7	29.22	303.05	−	+	N/A
(+)-Catechin ^a^	C15 H14 O6	17.51	289.07	−	+	N/A
Catechin (isomer) ^c^	C15 H14 O6	25.82	289.07	−	+	N/A
*Tannin*						
Procyanidin B-type dimer ^c^	C30 H26 O12	13.47	577.13	−	+	N/A

‘+’ = detected; ‘-’ = below limit of detection; ^a^ = identified based on mass spectrometer data and comparison of retention time with authentic standard; ^b^ = identified based on mass spectrometer data; ^c^ = identified based on mass spectrometer data and literature that confirm the presence in sorghum whole grains with authentic standard; ^d^ = comparison based on peak area if compounds are present in both samples; RT = retention time; *m/z* = mass-to-charge ratio; Y = yes; N = no (or equal); N/A = not applicable

### Study design and ethical aspects

A single-blind, randomized, placebo-controlled study was conducted at the Department of Nutrition and Health at the Federal University of Viçosa (UFV), Brazil, from April 2023 to September 2024. Those interested in participating in the study answered an online pre-screening questionnaire with questions about weight, height, medical history, and lifestyle habits. Based on the answers provided, volunteers were invited to attend an in-person assessment to verify the inclusion criteria.

The study included adults of both sexes, aged between 20 and 59 years, with body mass index (BMI) equal to or greater than 27.0 kg/m², body fat percentage higher than 30% for women and 20% for men, waist circumference higher than 80 cm for women and 92 cm for men, and physical activity level less than 150 min per week [17]. Smokers, frequent consumers of alcohol, pregnant or lactating women, people with a history of digestive, liver, kidney, cardiovascular, thyroid or recent inflammatory diseases, users of anti-inflammatory drugs and/or corticosteroids for continuous use, or who had used laxatives or antibiotics in the three months prior to the study were not included. Individuals who consumed probiotic, prebiotic or synbiotic products more than twice a week in the previous month, who had recent changes in physical activity level or who had eating disorders according to the Three-Factor Eating Behavior Questionnaire [18] were also not included.

A total of 51 volunteers were randomly assigned to one of two experimental groups: the sorghum group (SG) (*n* = 25) or the control group (CG) (*n* = 26). The allocation process was carried out using MinimPy software, version 2.0 [19], ensuring balance in factors such as sex, age, and body mass index. Volunteers underwent a nutritional intervention with a daily calorie reduction of 500 kcal and consumed their designated beverages over a ten-week period. In-person visits were conducted before and after the intervention period for analyze the anthropometric measurements, body composition, biochemical markers, and completion of the Bristol Stool Scale (BSS). Each volunteer also provided fecal samples before and after the intervention period.

This study was approved by the Human Research Ethics Committee of the Federal University of Viçosa (UFV) (5.162.838/ CAAE: 53827321.4.0000.5153 - November 24th, 2021) and registered in the Brazilian Clinical Trials Registry (REBEC) (registration number: RBR-32v2gm5 – Abril 05th, 2023). The ethical guidelines of the Declaration of Helsinki were followed, and informed consent was obtained from all individual participants included in the study. Participant confidentiality was maintained throughout the study. This study followed the CONSORT (Consolidated Standards of Reporting Trials) guidelines for reporting randomized clinical trials [20].

### Sample collection and analyzes performed

Weight and body fat percentage were measured using bioimpedance (Tanita BC-558 Ironman, Tokyo, Japan). Height was measured using a vertical anthropometer (Alturexata Ltd., Belo Horizonte, Brazil), and the waist circumference, hip circumference, and thigh circumference were measured in centimeters using a non-elastic tape. All measurements were performed again at the end of the study. Weight and height data were used to calculate the body mass index (BMI, kg/m²).

Venous blood samples were drawn after a 12-hour overnight fast into 4 mL vacuum tubes containing ethylenediaminetetraacetic acid (EDTA) and clot-activator gel, for the subsequent separation of plasma and serum, respectively. The blood was centrifuged at 2422 *g* at 4 °C for 15 min, and the concentrations of glycemia, triglycerides, uric acid, total cholesterol, high-density lipoprotein (HDL-c), and low-density lipoprotein (LDL-c) were determined using commercial Bioclin^®^ kits (Belo Horizonte, Brazil), following the manufacturer’s recommendations. Insulin and high-sensitivity C-reactive protein (CRP-*hs*) concentrations were analyzed in serum using chemiluminescence and immunoturbidimetry, respectively. Additionally, cardiometabolic risk index were calculated, such as Castelli Index I (total cholesterol/HDL-c) [21], Castelli Index II (LDL-c/HDL-c) [21], Homeostatic Model Assessment for Insulin Resistance (HOMA-IR) (glucose × insulin)/22.5 [22], and Triglyceride-Glucose Index (TyG index) (log (triglycerides × glucose) / 2) [23].

Each participant provided fecal samples in sterile and disposable hygienic containers. The feces were portioned into Eppendorf tubes and frozen at -80 °C until the time of analysis.

Dietary intake was evaluated using three 24-hour food records (two on weekdays and one on a weekend day) collected at two time points: baseline and the end of the intervention. Participants received detailed instructions from a trained dietitian on how to accurately complete the records, including guidance on estimating portion sizes using household measures and describing food preparation methods. All food records were reviewed in person with each participant to ensure accuracy, completeness, and clarity. Nutrient intake was analyzed using the Avanutri^®^ online software (Avanutri Assessment Equipment LTDA, Brazil), which is based on Brazilian food composition tables and supplemented with additional sources when necessary. Food intake was adjusted by body weight (g/kg) for caloric intake and macronutrients (carbohydrates, proteins, and fats), and by energy density (g/1000 kcal) for fiber. Dietary phenolic compounds were estimated using data from the United States Department of Agriculture (USDA) food composition databases [24–27]. The average daily intake was calculated at both time points (baseline and endpoint).

### Dietary intervention

Hypocaloric dietary plans were prescribed at the beginning of the study (500 kcal/day restriction) [28, 29]. The dietary plans were individualized after estimating the caloric needs of each volunteer (Estimated Energy Requirement – EER) according to weight, height, age and level of physical activity. The macronutrient distribution of the diets was similar among the volunteers (50% carbohydrates, 20% proteins, 30% lipids and 14 g of fiber for every 1,000 kilocalories), according to the Acceptable Macronutrient Distribution Range (AMDR) [30] (Supplementary Material 3).

### Blood metabolites

Stock solutions of naringenin, trans-caffeic acid, and protocatechuic acid were prepared in DMSO at concentrations of 1 mg/mL, 2 mg/mL, and 1 mg/mL, respectively, and subsequently diluted in methanol to obtain working solutions. Compound detection parameters were optimized via direct T-infusion at 1 µg/mL into the HPLC mobile phase (35% acetonitrile, 65% water with 0.1% formic acid) at a flow rate of 10 µL/min. The internal standard 4-hydroxy-3-nitrobenzoic acid (HNB) was prepared and optimized under the same conditions.

Plasma samples obtained from a subset of participants in the sorghum and control groups (*n* = 10/group) at the end of the intervention. Sample preparation was based on the method described by Chen et al. [31], with minor modifications. Briefly, 100 µL of plasma were transferred to a 1.5 mL microcentrifuge tube, followed by the addition of 2 µL of neat formic acid and 2.5 µL of internal standard (1 µg/mL in acetonitrile). Then, 1 mL of ethyl acetate was added, and the mixture was vortexed at 2500 rpm for 15 min at room temperature (25 °C). Samples were centrifuged at 21,130 ×*g* for 10 min, and 750 µL of the supernatant were transferred to a new tube and evaporated under nitrogen at 50 °C. The remaining samples were frozen at − 20 °C for at least 30 min, and any additional supernatant was collected and combined with the first fraction. Samples were dried completely and reconstituted in 100 µL of 50% acetonitrile containing 0.2% formic acid. After reconstitution, samples were vortexed, centrifuged, sonicated for approximately 12 min, and centrifuged again under the same conditions. Aliquots of 25 µL were transferred to autosampler vials for injections.

Calibration standards were prepared using 95 µL of blank plasma and 5 µL of mixed standard solution, resulting in final concentrations of 0.25, 1, 2.5, 10, 25, 50, and 125 ng/mL. LC-MS/MS analyses were performed using a Sciex 5500 QTRAP mass spectrometer coupled to an Agilent 1200 HPLC system equipped with a binary pump, thermostatted column compartment, and cooled autosampler. Chromatographic separation was achieved using a GL Sciences Inertsustain AQ-C18 column (2.1 × 150 mm, 3 μm), with mobile phases A (water with 0.1% formic acid) and B (acetonitrile with 0.1% formic acid). Data acquisition was performed in negative ion mode using multiple reaction monitoring (MRM). The column temperature was maintained at 35 °C, and the autosampler at 6 °C. A post-run re-equilibration time of 0.5 min was included. Calibration curves were generated using unweighted linear regression forced through the origin. The transition m/z 181.9 → 108.0 (HNB) was used as the internal standard to correct for extraction and reconstitution variability.

### Oxidative stress assessment

The Total plasma Antioxidant Capacity (TAC) was evaluated using the antioxidant assay kit (Sigma-Aldrich^®^, St. Louis, Missouri, USA), according to the manufacturer’s instructions, and the values were expressed in Trolox equivalents. Superoxide dismutase (SOD) activity was determined in serum, according to the method described by Marklund [32], based on the inhibition of pyrogallol oxidation, and enzymatic activity was expressed as SOD units (U). Catalase (CAT) activity was determined in serum using the colorimetric method described by Hadwan and Abed [33]. CAT activity was expressed in kilo Units per Liter (kU/L). Nitric oxide (NO) concentration was quantified in serum using the Griess method [34]. NO levels were expressed in µM. Lipid peroxidation was assessed by measuring malondialdehyde (MDA) levels in serum via the thiobarbituric acid reactive substances (TBARS) assay, according to Buege and Aust [35], and results were expressed in µM. All analyses were performed using a Varioskan Lux spectrophotometer (Thermo Fisher Scientifics; Waltham, MA, USA).

### Bristol stool scale, short-chain fatty acids content, and fecal pH

The Bristol Stool Scale (BSS) was used to obtain information about intestinal transit and function [36]. For this, the BSS was categorized into slow intestinal rhythm (types 1 and 2), adequate intestinal rhythm (types 3 and 4) and fast intestinal rhythm (types 5, 6 and 7).

Short-chain fatty acid (SCFA) analysis was performed using a 200 mg of feces dissolved in 2 mL of distilled water, with ethyl ether (1:1 v/v) using a vortex followed by centrifugation [37]. The determination was made by gas chromatography in the Agilent 7890 A equipment (Wilmington, DE, USA), using a FFAP capillary column (nitro terephthalic acid-modified polyethylene glycol, 25 m × 0.2 mm × 0.30 μm). The temperature was programmed to vary gradually from 40 to 230 °C, while the injector and detector were maintained at 250 and 280 °C, respectively. The identification of the compounds was performed using standards supplied by Sigma-Aldrich (St. Louis, MO, USA). The quantification was based on calibration curves, considering the relationship between the concentration and the relative area of the internal standard (crotonic acid) and different fatty acids. The results were expressed in mmol of fatty acid per kilogram of feces.

To measure fecal pH, 0.1 g of feces were diluted in deionized water (pH = 6.62) at a concentration of 0.1 g/mL [38] and fecal pH was measured using a digital pH meter pH-1900 (Instrutherm^®^, São Paulo, SP, Brazil).

### Statistical analysis

The Shapiro–Wilk test was performed to verify the normality of each variable, and the data are presented as means and standard deviations, and median and percentiles (p25-p75), bar graphs, box-and-whisker plots, and heatmap. The independent t-test and the Mann-Witney test were for used inter-group comparisons, while the paired t-test and the Wilcoxon test were used for intra-group comparisons. Spearman correlation analysis was performed to evaluate the relationship between sorghum intake, fecal pH, TAC, SOD, CAT, NO, MDA gastrointestinal symptoms, short-chain fatty acids content. Statistical analyses were performed using GraphPad Prism version 10.4.1, with significance level of *p* < 0.05.

The statistical power of the study was calculated using the G*Power 3.1 software [39], based on the difference between two independent means (two groups) and using a post hoc test. For the calculation, the values of total plasma antioxidant capacity and the number of volunteers in each group were used. An effect size of at least 0.71 and a significance level of 5% were applied, resulting in a statistical power of 80.3%.

## Results

### Characteristics of the participants

**Fig. 1:**
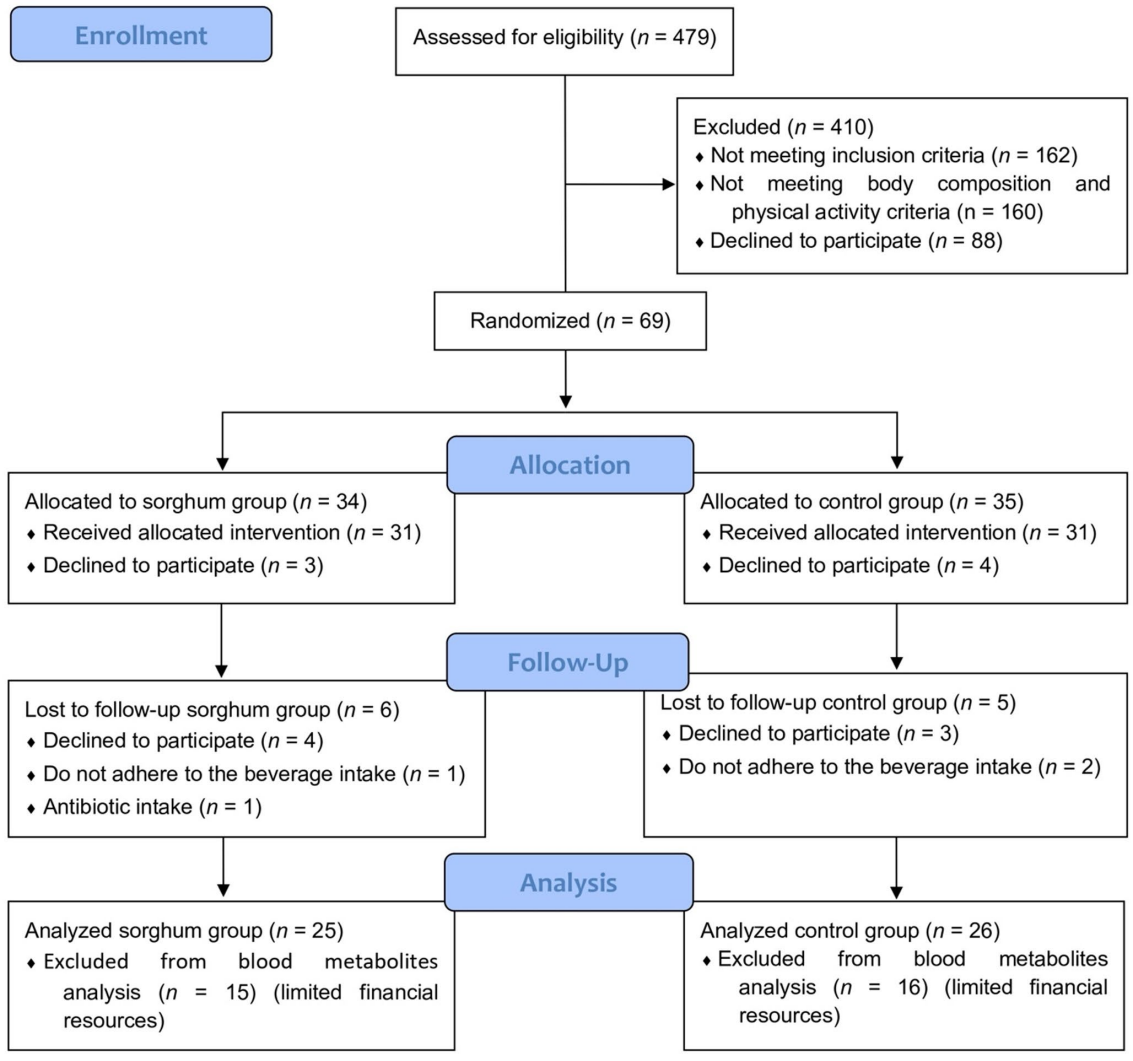
A CONSORT flow diagram of the participants

A total of 479 individuals completed the online pre-screening questionnaire. After eligibility screening, 162 were excluded for not meeting the inclusion criteria, and 88 were excluded after failing to respond to follow-up contact or withdrawing interest. During in-person assessments, 160 volunteers were excluded due to BMI < 27 kg/m², body fat below the established threshold, or high physical activity levels. A final sample of 69 participants was randomized into the sorghum group (SG, *n* = 34) or the control group (CG, *n* = 35). During the intervention, 18 participants (9 from each group) were excluded due to antibiotic use, withdrawal, or non-adherence to the beverage protocol. Thus, 51 participants completed the study (SG = 25; CG = 26) (Fig. 1).

The baseline characteristics of the final sample showed no differences between groups. The average age was similar (SG: 37.08 ± 9.92, CG: 38.31 ± 9.12; *p* = 0.647), and the gender distribution included 80% (*n* = 20) females in the SG and 84.6% (*n* = 22) in the CG.

### Food intake

Regarding dietary intake, although baseline differences were observed in protein, fat, fiber, and caloric intake between groups, no differences were found at the endpoint after ten weeks of dietary prescription and adjustment (*p* > 0.05). No differences were observed between groups for dietary phenolic compound intake at baseline, endpoint, or changes over time (delta) (*p* > 0.05). However, the delta differed between groups for fat (*p* = 0.035) and dietary fiber intake (*p* = 0.028) (Table 3).

**Table 3:** Dietary intake of nutrients and estimated phenolic compounds at baseline, endpoint, and delta in the control and sorghum groups

Outcomes	Baseline		*p*-value	Endpoint		*p*-value	Delta		*p*-value
	CG	SG		CG	SG		CG	SG	
Carbohydrate (g/kg BW)	2.83±0.95	3.08±1.05	0.382	2.45±0.90	2.36±0.93	0.739	-0.38±0.87	-0.72±0.90	0.187
Protein (g/kg BW)	0.89±0.20	1.15±0.31	**0.002**	0.87±0.29	1.02±0.32	0.089	-0.02±0.30	-0.13±0.30	0.231
Fat (g/kg BW)	0.73±0.21	1.02±0.40	**0.004**	0.60±0.25	0.67±0.29	0.364	-0.13±0.29	-0.34±0.37	**0.035**
Dietary fiber (g/1000 kcal)	12.47±4.56	9.40±4.16	**0.020**	12.84±3.71	12.94±5.33	0.945	0.37±4.93	3.54±4.60	**0.028**
Caloric intake (kcal/kg BW)	21.42±5.60	26.07±8.23	**0.031**	18.65±6.58	19.79±7.08	0.603	-2.77±6.55	-6.36±7.37	0.086
Proanthocyanidin (mg)	260.50±279.36	256.89±257.60	0.728	316.03±347.82	348.48±291.76	0.482	55.53±389.77	91.59±227.30	0.358
Isoflavones (mg)	0.20±0.16	0.21±0.54	0.053	0.13±0.08	0.10±0.09	0.187	-0.13±0.31	-0.10±0.54	0.099
Flavan-3-ols (mg)	6.83±5.75	8.35±9.04	0.981	4.64±4.27	9.03±11.78	0.481	-2.19±3.60	0.68±10.74	0.091
Flavones (mg)	0.29±0.43	0.30±0.41	0.999	0.33±0.44	0.28±0.39	0.974	0.04±0.68	-0.02±0.56	0.758
Flavonols (mg)	9.08±5.27	8.02±6.37	0.171	7.92±4.99	10.02±5.20	0.078	-1.15±7.60	2.00±4.95	0.194
Flavanones (mg)	13.12±25.15	10.64±22.63	0.624	6.92±18.80	16.98±34.60	0.176	-6.19±29.38	6.34±44.53	0.908
Anthocyanidins (mg)	5.26±13.94	9.68±20.47	0.897	1.33±2.74	3.38±8.50	0.820	-3.93±14.14	-6.30±22.79	0.766
Phenolic acids (mg)	184.94±132.76	148.75±98.31	0.293	170.97±114.34	126.13±91.65	0.144	-13.97±103.72	-22.62±84.10	0.754
Total estimated phenolic compounds (mg)	479.22±2279.44	442.16±281.99	0.653	507.52±367.26	393.72±282.20	0.239	27.12±398.60	-48.44±287.63	0.458

CG = control group; SG = sorghum group; Delta = endpoint – baseline; g/kg BW = grams per kilogram of body weight; kcal/kg BW = kilocalorie per kilogram of body weight; kcal = kilocalorie

### Effects of sorghum and control beverages on plasma phenolic metabolites, total plasma antioxidant capacity and oxidative stress

**Fig. 2:**
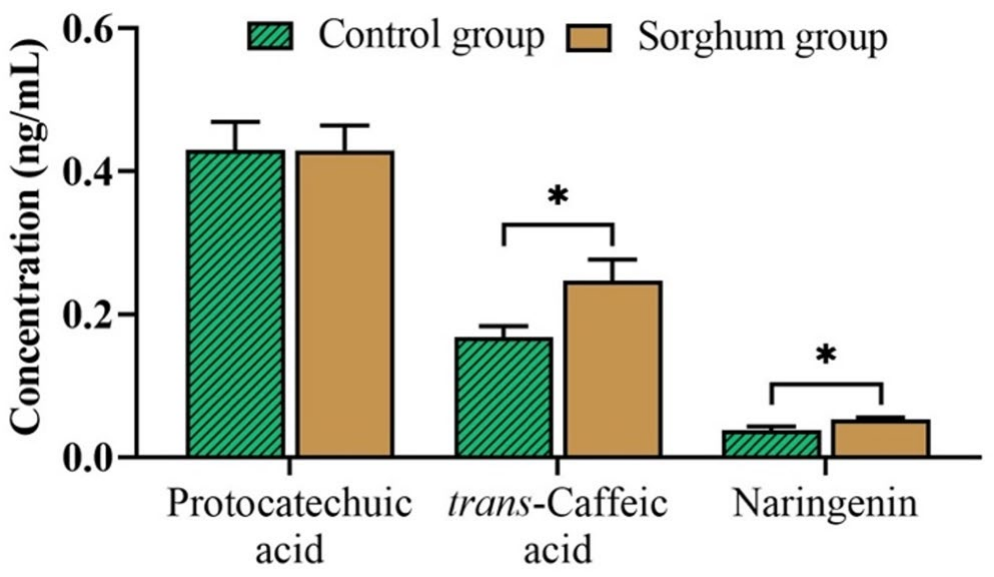
Plasma phenolic metabolites after sorghum and control beverages consumption. Data are presented as media and SD. Intergroup comparisons of endpoint values were analyzed using the independent t-test. Statistical significance is indicated as follows: * *p* < 0.05

Regarding plasma phenolic metabolites after the intervention, SG exhibited higher plasma concentrations of *trans*-caffeic acid (*p* = 0.032) and naringenin (*p* = 0.027) compared to CG (Fig. 2). In contrast, no difference was observed in protocatechuic acid levels between groups (*p* = 0.993).

In the comparison of total plasma antioxidant capacity (TAC) (intra-group), SG increased TAC at the endpoint compared to baseline (*p* = 0.009) (Table 3). No changes were observed in the TAC of CG (*p* = 0.555). Furthermore, in the comparison of TAC variation (delta) inter-group, SG increased TAC (positive delta) compared to CG (*p* = 0.023), which decreased TAC at the end of the intervention (negative delta). For superoxide dismutase (SOD) (intra-group), SG decreased SOD at the endpoint compared to baseline (*p* = 0.039). No changes were observed in the SOD of CG (*p* = 0.079) nor in the comparisons inter-groups or delta. Further, SG decreased catalase (CAT) (negative delta) compared to CG (*p* = 0.037), who increased CAT at the end of the intervention (positive delta). No changes were observed inter-group and intra-group in the CAT (*p* > 0.05). Furthermore, for nitric oxide (NO), CG decreased at the endpoint compared to baseline (*p* = 0.036). No changes were observed in NO in the SG (*p* > 0.05); and for malondialdehyde (MDA), both the SG (*p* = 0.012) and the CG (*p* = 0.003) decreased at the endpoint compared to baseline. However, no differences were observed in the inter-group (*p* > 0.05) (Table 4).

**Table 4:** Total plasma antioxidant capacity and oxidative stress of adults with excess body weight at baseline and after consuming control or sorghum beverages, with delta values

Outcomes	CG	SG	*p*-value (inter-group)
TAC			
Baseline	0.93±0.19 ^a^	0.85±0.15 ^b^	0.151
Endpoint	0.91±0.16 ^a^	0.94±0.22 ^a^	0.550
Delta	−0.02±0.16	0.09±0.15	**0.023**
SOD (U)			
Baseline	3.32±0.76 ^a^	3.34±0.63 ^a^	0.463
Endpoint	3.05±0.42 ^a^	3.07±0.56 ^b^	0.927
Delta	−0.27±0.59	−0.36±0.71	0.386
CAT (kU/L)			
Baseline	41.94±25.81 ^a^	53.82±3242 ^a^	0.153
Endpoint	53.16±33.26 ^a^	43.53±24.62 ^a^	0.247
Delta	11.22±41.85	−10.29±28.47	**0.037**
NO (µM)			
Baseline	1.43(0.23–3.49) ^a^	1.58(0.11–3.56) ^a^	0.897
Endpoint	0.48(0.00–2.15) ^b^	0.90(0.00–2.45) ^a^	0.685
Delta	−0.59(−2.53–0.24)	−0.23(−2.20–0.08)	0.955
MDA (µM)			
Baseline	1.12±0.15 ^a^	1.09±0.17 ^a^	0.540
Endpoint	0.99±0.19 ^b^	0.99±0.22 ^b^	0.999
Delta	−0.13±0.18	−0.10±0.16	0.551

Data are presented as mean ± SD and median and percentiles (p25-p75). Intergroup comparisons (CG vs. SG) are presented in the rows, whereas intragroup comparisons (baseline vs. endpoint) are presented in the columns. Intragroup differences were assessed using a paired t-test or Mann-Witney test, and intergroup differences were assessed using an independent t-test or Wilcoxon test. A p-value ≤ 0.05 was considered significant. Intergroup differences are indicated in bold within the columns, while intragroup differences are indicated by different letters within the rows. CG = control group; SG = sorghum group; TAC = Total plasma Antioxidant Capacity; SOD = Superoxide dismutase; CAT = Catalase; NO = Nitric oxide; MDA = Malondialdehyde; delta = endpoint − baseline.

### Effects of sorghum and control beverages on anthropometry, body composition, biochemical markers, and cardiovascular risk index

Volunteers in both groups presented no differences in body weight, body mass index (BMI) and circumferences in either intra-group or inter-group (*p* > 0.05), except for body fat percentage, which was higher in CG at the endpoint compared to baseline (*p* = 0.008) (Table 5).

**Fig. 3:**
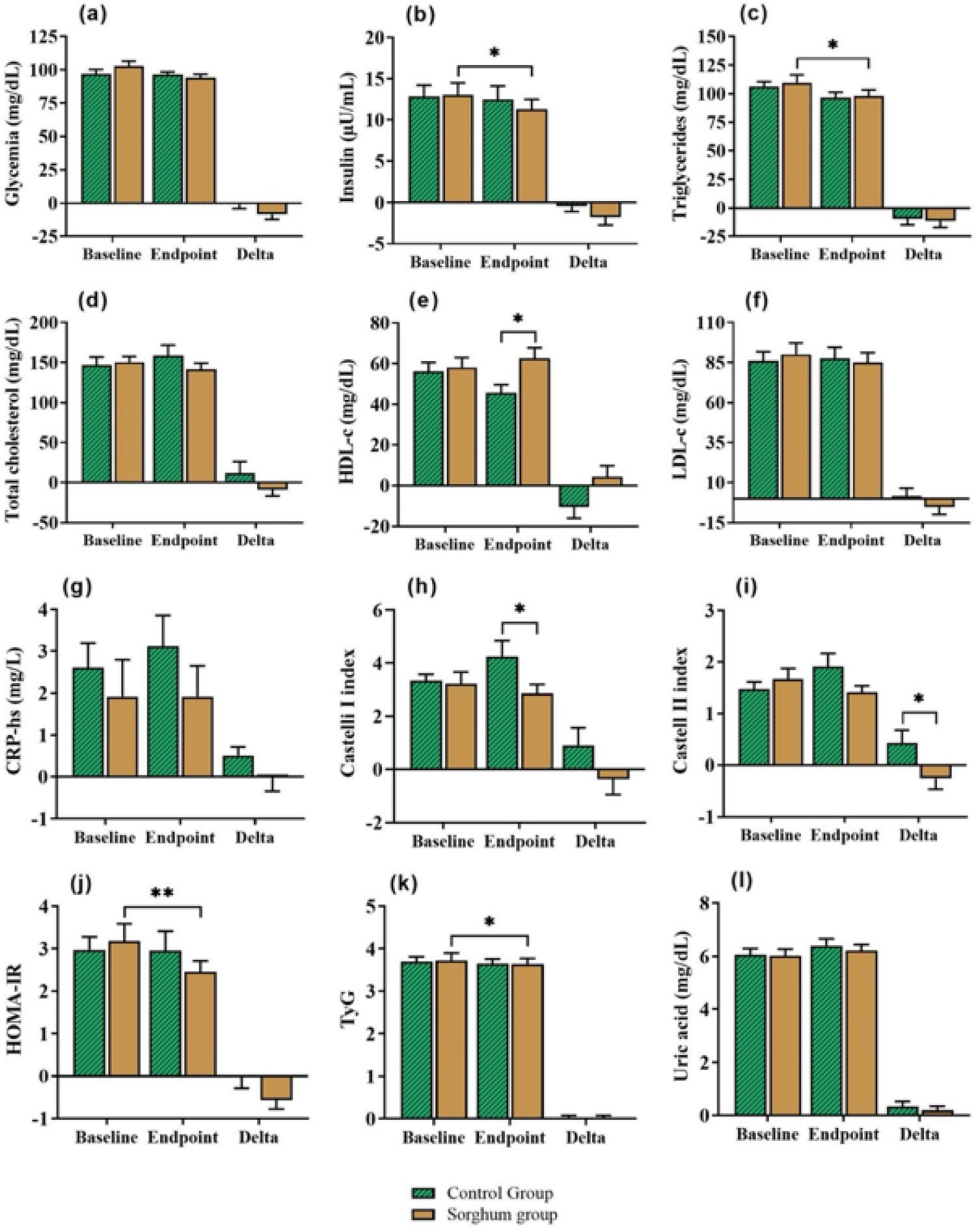
Biochemist marks of adults with excess body weight at baseline and after consuming control or sorghum beverages, with delta values. Glycemia **a**; Insulin **b**; Triglycerides **c**; Total cholesterol **d**; HDL-c = high-density lipoprotein **e**; LDL-c = low-density lipoprotein **f**; CRP-*hs* = high-sensitivity C reactive protein **g**; Castelli I index = total-cholesterol/HDL-c **h**; Castelli index II = LDL-c/HDL-c **i**; HOMA-IR = (glycemia x insulin) / 22,5 **j**; TyG index = (log (triglycerides x glycemia) /2) **k**; Uric acid **l**; Delta = endpoint – baseline. * *p* < 0.05; ** *p* < 0.01. The normal reference values used to interpret metabolic markers based on established clinical guidelines: Fasting glycemia: <100 mg/dL; Insulin: ≤25 µU/mL; Triglycerides: <150 mg/dL; Total cholesterol: <200 mg/dL; HDL-c: ≥40 mg/dL for men and ≥ 50 mg/dL for women; LDL-c: <100 mg/dL; CRP-*hs*: <1.0 mg/L indicating low risk, 1.0–3.0 mg/L moderate risk, and > 3.0 mg/L high risk; Castelli I Index I: <4.5 for men and < 4.0 for women; Castelli Risk Index II: <3.3 for men and < 3.0 for women; HOMA-IR: >2.5 suggest reduced insulin sensitivity; TyG index: elevated when > 8.7; Uric acid: elevated when > 7.0 mg/dL in men and > 6.0 mg/dL in women

Regarding biochemical markers, in inter-group at endpoint, SG exhibited higher HDL-c (*p* = 0.018), and lower Castelli I Index (*p* = 0.012) compared to the CG (Fig. 3e and h). Furthermore, when analyzing delta, SG decreased Castelli II Index (*p* = 0.031), and CG increased this index (Fig. 3i). Furthermore, SG improved several parameters from baseline. Specifically, reductions in insulin (*p* = 0.047), triglycerides (*p* = 0.041), HOMA-IR (*p* = 0.008), and TyG index (*p* = 0.016) were observed at the endpoint (Fig. 3b-c, j-k).

**Table 5:** Anthropometry, body composition, adiposity indices and cardiovascular risk indices of adults with excess body weight at baseline and after consuming control or sorghum beverages, with delta values

Outcomes	CG	SG	*p*-value (inter-group)
Body weight (kg)			
Baseline	82.79±10.3 ^a^	83.52±14.82 ^a^	0.980
Endpoint	82.60±10.95 ^a^	83.81±15.32 ^a^	0.753
Delta	−0.19±2.19	0.29±2.07	0.429
Body fat (%)			
Baseline	39.15±7.53 ^b^	38.32±5.38 ^a^	0.303
Endpoint	41.40±6.96 ^a^	39.13±5.61 ^a^	0.316
Delta	2.25±4.02	0.81±2.85	0.502
BMI (kg/m^2^)			
Baseline	31.54±4.20 ^a^	31.05±3.59 ^a^	0.693
Endpoint	31.46±4.41 ^a^	30.98±3.40 ^a^	0.675
Delta	−0.08±0.84	−0.07±0.81	0.428
Waist circumference (cm)			
Baseline	102.10±11.15 ^a^	98.77±11.07 ^a^	0.277
Endpoint	100.50±11.97 ^a^	97.43±10.95 ^a^	0.349
Delta	−1.60±4.76	−1.34±3.97	0.842
Hip circumference (cm)			
Baseline	113.80±11.12 ^a^	111.10±7.88 ^a^	0.620
Endpoint	112.80±10.97 ^a^	110.10±7.91 ^a^	0.322
Delta	−1.00±4.27	−1.00±3.17	0.689
Thigh circumference (cm)			
Baseline	60.21±7.15 ^a^	58.51±4.42 ^a^	0.322
Endpoint	60.49±7.87 ^a^	58.52±4.32 ^a^	0.336
Delta	0.28±3.86	0.01±2.60	0.772

Data are presented as mean ± SD. Intergroup comparisons (CG vs. SG) are presented in the rows, whereas intragroup comparisons (baseline vs. endpoint) are presented in the columns. Intragroup differences were assessed using a paired t-test, and intergroup differences were assessed using an independent t-test. A p-value ≤ 0.05 was considered statistically significant. Significant intergroup differences are indicated in bold within the columns, while significant intragroup differences are indicated by different letters within the rows. CG = control group; SG = sorghum group; BMI = body mass index; delta = endpoint − baseline.

### Effects of sorghum and control beverages on stool consistence, short-chain fatty acids content, and fecal pH

Stool consistency, evaluated using the Bristol Stool Scale (BSS) at baseline, revealed that 84% (*n* = 21) of the SG and 57.8% (*n* = 15) of the CG exhibited type 3 or 4 stools, classified as normal. Post-intervention, the prevalence of normal stool types increased to 92% (*n* = 23) in the SG and 61.6% (*n* = 16) in the CG.

**Fig. 4:**
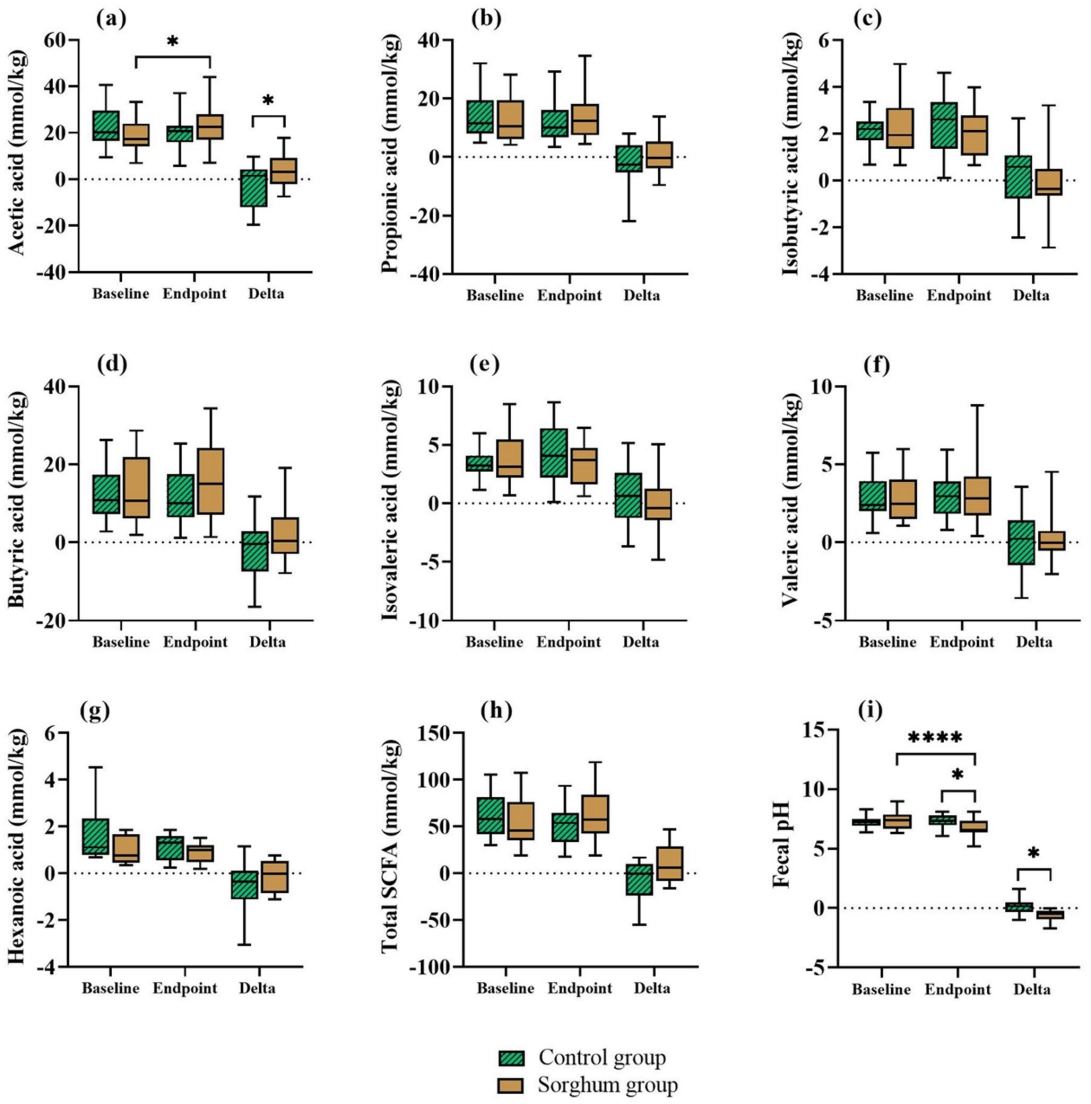
Short-chain fatty acid **a-h**, and fecal pH **i** of adults with excess body weight at baseline and after consuming control or sorghum beverages, with delta values. The horizontal line within each box represents the median, the lower and upper bounds of the box represent the first and third quartiles, respectively, and the lower and uppr error bars represent the minimum and maximum values. Intragroup comparisons between baseline and endpoint were performed using the paired t-test or the Wilcoxon test, and intergroup comparisons of baseline, endpoint, and delta values were analyzed using the independent t-test or the Mann-Witney test. Statistical significance is indicated as follows: * *p* < 0.05; **** *p* < 0.0001. GSRS = Gastrointestinal Symptoms Rating Scale; Delta = endpoint – baseline

Regarding the short-chain fatty acids (SCFA), SG increased acetic acid at the end of the intervention (positive delta) compared to CG (*p* = 0.024), who decreased at the end of the intervention (negative delta). Furthermore, SG increased acetic acid at the endpoint compared to baseline (*p* = 0.025) (Fig. 4a). No changes were observed in acetic acid within the CG group (*p* = 0.323), nor in the other SCFA (*p* > 0.05).

Moreover, SG decreased fecal pH at the endpoint compared to CG (*p* = 0.015), and delta (*p* < 0.0001). Similarly, SG decreased fecal pH intra-group at the endpoint compared to baseline (*p* < 0.0001) (Fig. 4i).

### Correlation of sorghum intake with blood metabolites, total plasma antioxidant capacity and oxidative stress, and intestinal health

**Fig. 5:**
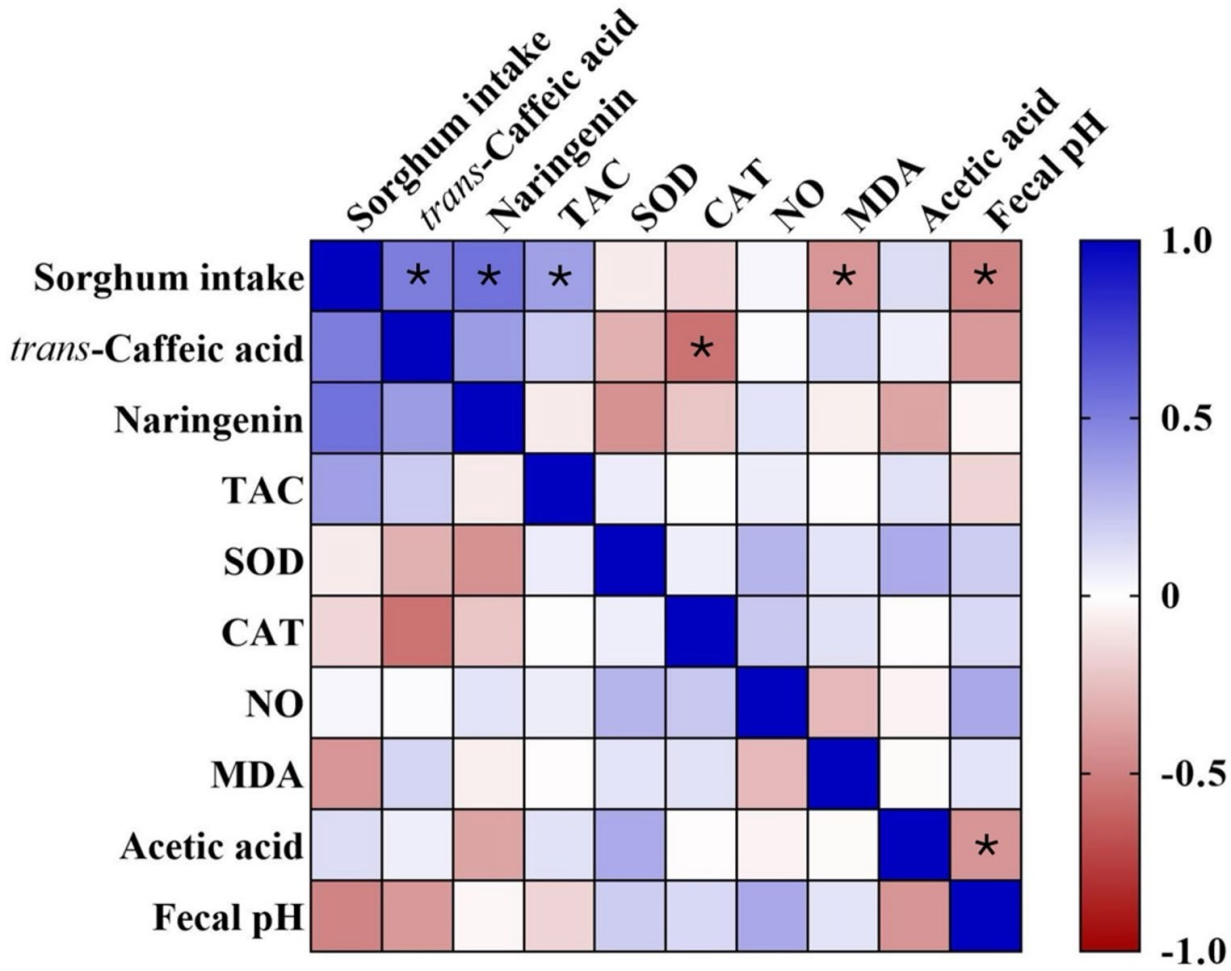
Heatmap of Spearman’s correlations between sorghum intake (grams of sorghum intake/kg of body weight), blood metabolites, total plasma antioxidant capacity (TAC), superoxide dismutase (SOD), catalase (CAT), nitric oxide (NO), malondialdehyde (MDA) acetic acid and fecal pH. * *p* < 0.05

When evaluating the correlations between sorghum intake, blood metabolites, total plasma antioxidant capacity, oxidative stress and intestinal parameters, sorghum intake presented a positive correlation with *trans*-caffeic acid (*r* = 0.51; *p* = 0.022), naringenin (*r* = 0.55; *p* = 0.012) and TAC (*r* = 0.37; *p* = 0.010), and a negative correlation with MDA (*r* = −0.41; *p* = 0.007) and fecal pH (*r* = −0.49; *p* = 0.001 ). Fecal pH also was negatively correlated with acetic acid (*r* = −0.42; *p* = 0.010). Furthermore, *trans*-caffeic acid presented a negative correlation with CAT (*r* = −0.55; *p* = 0.013) (Fig. 5).

## Discussion

By integrating clinical, biochemical, and metabolomic outcomes, this study provides novel insights into the role of sorghum phenolics on fecal and blood metabolites, oxidative balance, and cardiometabolic parameters in adults with excess body weight, an important intervention point given the increasing global prevalence of obesity. Although differences in energy and macronutrient intake were observed between the control group (CG) and the sorghum group (SG) at baseline, dietary intake became comparable between groups at the endpoint following dietary prescription and monitoring.

The maintenance of comparable phenolic compound intake between groups in baseline and endpoint can be explained by the wide distribution of phenolic compounds across commonly consumed foods, such as fruits, vegetables, cereals, coffee, and legumes [24–27] and by standardized dietary prescription. Although the baseline differences in the intake of macronutrients were observed between the CG and SG groups, the implementation of dietary adjustments occurs during the intervention time, demonstrating a reduction in fat and an increase in dietary fiber intake in SG group, since sorghum beverage provide more dietary fiber content. However, fat intake became similar at the endpoint, mainly due to a higher reduction in fat intake in the SG, as confirmed by delta analysis.

Although participants in both groups were prescribed an energy-restricted diet (~ 500 kcal/day deficit), no significant changes in body weight, BMI, or circumferences were observed over the 10-week intervention. This finding may be explained by metabolic adaptation, that may reduce the energy expenditure and to attenuate weight loss. Further, other environment aspects can affect the body weight outcomes, such as follow the dietary prescriptions and physical activity levels [40]. Body weight alone may not fully reflect metabolic improvements, as evidenced by the favorable changes observed in the SG, including reductions in insulin and triglycerides levels, HOMA-IR, TyG index, and Castelli II Index, as well as higher HDL-c at the endpoint. The higher levels of HDL-c levels in the SG may be related to the bioactive profile of sorghum, particularly its phenolic compounds, such as protocatechuic acid and trans-caffeic acid. These compounds exert antioxidant effects that may protect HDL from oxidative damage and contribute to preventing the decline in HDL-c, even under energy-restricted condition [41].These findings suggest that improvements in metabolic health can occur independently of significant changes in body weight, potentially mediated by alterations on dietary composition, such as increase of dietary fiber intake and decrease of fat intake.

For this study, the BRS 305 genotype was selected by Sorghum genetic improvement program of *Embrapa Milho e Sorgo* due to its high content of bioactive compounds with potential health benefits [42, 43]. The extruded BRS 305 sorghum-based beverage stood out for its richness in phenolic acids, such as protocatechuic acid and *trans*-caffeic acid, as well as flavonoids like naringenin and catechin, and condensed tannins such as procyanidin B-type dimer. High concentrations of bound phenolic acids such as caffeic acid (39.65 µg/g), p-coumaric acid (121.40 µg/g), and ferulic acid (529.00 µg/g) were previously identified in extruded BRS 305 sorghum, further demonstrating the presence of relevant hydroxycinnamic acids and reinforcing the robustness of its phenolic profile after extrusion [44]. Most of these compounds were found in higher concentrations or exclusively in the sorghum beverage. Compounds that did not differ between beverages are likely present in soy milk [45], which was used in the preparation of both beverages. Although the apples and grapes are source of phenolic compounds such as 5-caffeoylquinic acid, (+)-catechin, and procyanidin B-type dimers, the compounds were not detected in the control beverage added with these fruit juices. This finding may be explained by matrix effects and processing-related factors, including dilution, oxidation, thermal degradation, and interactions with dietary fibers and other phenolics, which can impair compound stability and analytical detectability. Consistent with this interpretation, previous studies have reported substantial matrix- and processing-related losses of phenolic compounds in complex fruit-based beverages [46, 47].

The high total phenolic content observed in the sorghum beverage, as determined by the Folin–Ciocalteu assay, differed substantially from values previously reported for extruded whole-grain sorghum flour from the same research group [44]. Since both studies employed the same extrusion conditions, these discrepancies are more likely explained by differences in the food matrix, the relative contribution of free and bound phenolic fractions, and particularly the extraction methods used. In the present study, phenolic compounds were extracted from lyophilized beverages using a methanol: water solution (60:40, v/v) with a lower solvent-to-sample ratio (10:1) and an extraction time of 16 h, followed by centrifugation at 1,509 × g for 10 min. In contrast, the previous study used 80% ethanol with a substantially higher solvent-to-sample ratio (100:1) and an extraction time of 12 h, followed by centrifugation at 1,800 × g for 5 min. Differences in solvent polarity, solvent-to-sample ratio, extraction conditions, and sample matrix may considerably affect the extraction efficiency and recovery of phenolic compounds. Methanol has a higher polarity than ethanol, which may enhance the extraction of low-molecular-weight phenolics [48] and partially explain the higher total phenolic concentration observed in the present study. In addition, the longer extraction time in the present study may have favored greater extraction. It is also important to note that the Folin–Ciocalteu method is not specific to phenolic compounds and may detect other reducing substances, potentially contributing to an overestimation of total phenolic content.

**Fig. 6:**
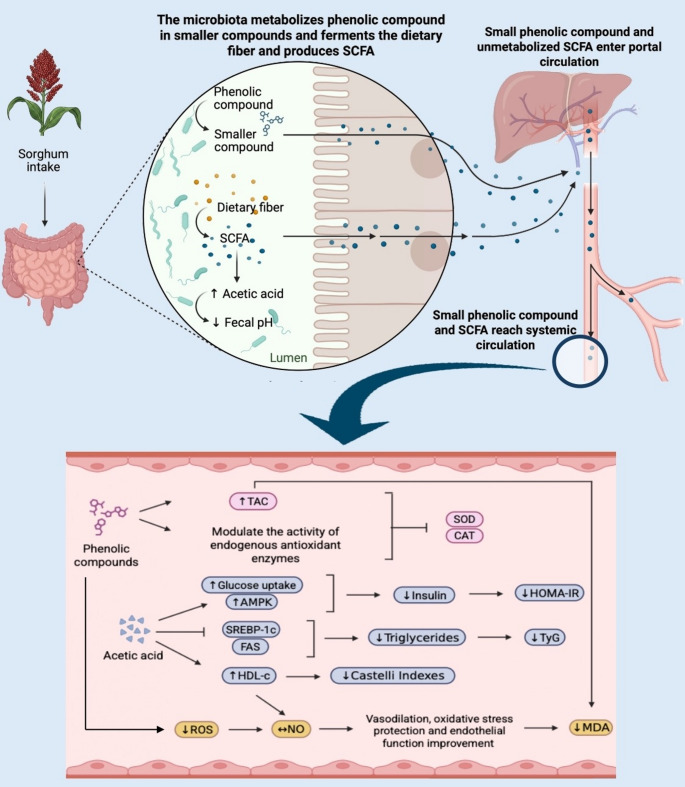
Summary of the main metabolic pathways and mechanisms modulated by sorghum intake. SCFA: short chain fatty acid; TAC: total plasma antioxidant capacity; SOD: superoxide dismutase; CAT: catalase; AMPK: AMP-activated protein kinase; HOMA-IR: homeostatic model assessment for insulin resistance; SREBP-1c: sterol regulatory element-binding protein-1c; FAS: fatty acid synthase; TyG: triglyceride-glucose index; HDL-c: high-density lipoprotein; ROS: reactive oxygen species; NO: nitric oxide; MDA: malondialdehyde. Created in BioRender. Aguiar, L. (2025) https://BioRender.com/6so2a3p and https://BioRender.com/6h7kybu

It is estimated that only 5–10% of phenolic compounds, intake through food, are absorbed [49]. Smaller compounds can be absorbed in the small intestine, and more complex compounds, such as tannins, are metabolized by the gut microbiota into smaller molecules in the colon, which can, then, be absorbed and reach systemic circulation [50–52]. The metabolic pathways and mechanisms discussed throughout this section are summarized in Fig. 6. This schematic representation provides an integrated overview of the effects observed, highlighting the interactions between lipid metabolism, glycemic control, antioxidant responses, and endothelial function in the context of sorghum intake.

A higher concentration of phenolic compounds was detected in the plasma of the SG, indicating that sorghum beverage intake increased systemic availability of these compounds, likely due to their metabolism and absorption. This hypothesis is supported by the statistical findings, where 26% of the variation in trans-caffeic acid (*r²* = 0.26) and 30% of naringenin (*r²* = 0.30) were explained by beverages sorghum intake. Moreover, *trans*-caffeic acid can be formed as the result of microbial or digestive metabolites of more complex phenolics such as 5-caffeoylquinic acid, ferulic acid, and p-coumaric acid, found in sorghum [53].

The phenolic compounds detected in plasma contributed directly to the total antioxidant capacity (TAC), as evidenced by the association between their concentrations and TAC. Sorghum intake explained approximately 14% of the variation in plasma TAC (*r*² = 0.137), suggesting that phenolic compounds present in the beverage enhanced systemic antioxidant defenses. These compounds, such as phenolic acids and flavonoids, act by neutralizing reactive oxygen species (ROS), chelating transition metal ions, and modulating the activity of endogenous antioxidant enzymes like superoxide dismutase (SOD) [54]. Interestingly, despite the well-established antioxidant potential of phenolics, SG showed reduced activity of endogenous enzymes, including SOD and catalase (CAT). This reduction likely reflects a compensatory physiological mechanism, whereby the presence of potent exogenous antioxidants from sorghum reduces the need for enzymatic antioxidant defenses [55] (Fig. 6). Supporting this, trans-caffeic acid alone explained 30% of the variation in CAT activity (*r*² = 0.30), and the negative correlation observed indicates that higher levels of this compound were associated with lower CAT activity. These findings reinforce the hypothesis that phenolics derived from sorghum not only increase antioxidant capacity directly but also modulate endogenous responses by lowering oxidative stress.

Although phenolic compounds are not directly fermented into short-chain fatty acids (SCFA), they can modulate the composition and metabolic activity of the gut microbiota by selectively promoting the growth of beneficial bacteria, including SCFA-producing bacteria [56]. In the present study, SG increased fecal acetic acid, a major SCFA, which was associated with a reduction in fecal pH and improved stool consistency. These effects suggest an acidification of the intestinal lumen, which may contribute to a more favorable microbial environment [57]. Sorghum intake explained 23.6% of the variation in fecal pH (*r*² = 0.236), while acetic acid content alone accounted for approximately 18% of this variation (*r*² = 0.176). These findings support the hypothesis that BRS 305 sorghum, as a whole food matrix rich in phenolic compounds modulates gut microbial metabolism and contributes to intestinal health by enhancing SCFA production and improving luminal conditions.

Moreover, the increase in acetic acid may enhance insulin sensitivity by promoting glucose uptake in peripheral tissues and activating AMP-activated protein kinase (AMPK), a key regulator of energy homeostasis that favors glucose metabolism [58, 59]. In SG, the insulin levels decreased, and markers of insulin sensitivity improved, as indicated by lower HOMA-IR and TyG index values. These findings suggest that sorghum intake, possibly through the production of acetic acid and other fermentation-derived metabolites, may contribute to improve metabolic functions by modulating key pathways involved in insulin signaling.

Acetic acid has been associated with the modulation of lipid metabolism, mainly by reducing hepatic lipogenesis and inhibiting genes involved in fatty acid and triglyceride synthesis, such as fatty acid synthase (FAS) and sterol regulatory element-binding protein-1c (SREBP-1c) [60]. Some evidence also suggests that acetic acid may modestly increase HDL-c levels, which is beneficial due to its role in reverse cholesterol transport and protection against atherosclerosis [61].

Additionally, nitric oxide (NO) concentrations were preserved. HDL-c is known to stimulate NO production, thereby promoting vasodilation and protecting against oxidative stress [62]. Phenolic compounds have been associated with improved endothelial function through mechanisms related to the reduction of oxidative stress and increased NO bioavailability [63]. These vascular protective effects are supported by the reduction in plasma levels of malondialdehyde (MDA), a well-established biomarker of oxidative stress and lipid peroxidation [64]. Although a reduction in MDA was observed in both groups, this finding is likely related to overall dietary adjustments made during the intervention period. Notably, SG exhibited a more consistent pattern of improvement across multiple parameters, including oxidative stress markers, antioxidant capacity, and biochemical indicators. These findings suggest that the sorghum beverage contributed to a more favorable metabolic and oxidative profile, although the specific bioactive compounds underlying these effects cannot be identified.

Additionally, SG maintained body composition, suggesting that phenolic compounds in SG may have contributed to body fat regulation, since the CG increased the body fat percentage. Studies show that regular phenolic intake can reduce visceral fat accumulation, which is closely linked to cardiovascular diseases and metabolic syndrome [65, 66]. In pilot study [16], a reduction in estimated total visceral fat was observed in the SG, while the CG increased, supporting the body composition benefits of the sorghum beverage. Notably, the increase in body fat observed in CG in the present study appears to be associated with visceral fat, as no significant changes were found in body circumferences. Although phenolic compounds are often highlighted for their potential metabolic benefits, it is essential to consider the overall food matrix. Sorghum beverage provides a complex nutritional profile, including dietary fibers, resistant starch, and a variety of bioactive compounds such as tannins and phenolic acids, which likely act synergistically to influence metabolic outcomes. This interaction within the food matrix is more relevant than the isolated effect of a single compound, as it reflects how these components are digested, metabolized, and absorbed in vivo.

This study presents strengths, including its randomized, placebo-controlled, single-blind design, which minimizes bias and enhances the validity of the findings; the use of whole-grain sorghum beverages formulated with BRS 305, a phenolic-rich hybrid, adds originality and relevance, considering the growing interest in plant-based functional foods; a comprehensive evaluation was conducted, integrating systemic (plasma) and intestinal (fecal) outcomes to explore biochemical, metabolic, and gut health markers; and the simultaneous analysis of bioactive compounds in the beverages and in biological samples, supported the correlation between intake and systemic availability, offering valuable insights into the bioaccessibility and potential mechanisms of action of phenolic compounds. Besides, both sorghum-based and control beverages contained identical amounts of soymilk and fruit juices, ensuring a comparable contribution of these ingredients to the polyphenol content between groups. Despite these strengths, some limitations should be acknowledged: the 10-week duration, although sufficient to detect short-term effects, may not reflect the long-term impact of sorghum intake on metabolic alterations; blood metabolites were analyzed in only 10 volunteers from each group at the end of the intervention, rather than in the full sample; and only three metabolites were quantified in plasma, although several other compounds were identified in the beverages. The present study also has some limitations regarding the chemical characterization of phenolic compounds in the beverages. Although the Folin–Ciocalteu assay indicated a substantial increase in total phenolic compounds in the sorghum beverage and the UHPLC analysis presented the qualitative profile of sorghum and control beverages, quantitative data on individual phenolics were not obtained. This limitation should be considered when interpreting the results, and future studies are encouraged to include detailed profiling of individual phenolic compounds to better elucidate the observed differences.

## Conclusion

The extruded BRS 305 sorghum-based beverage, rich in phenolic compounds including *trans*-caffeic acid and naringenin, improved acetic acid content, blood metabolites, oxidative balance, and cardiometabolic markers in adults with excess body weight. The application of a real-food matrix such as whole sorghum, cultivated and consumed globally, enhances the translational potential of the results and reinforces their relevance for public health recommendations and dietary guidelines.

## Supplementary Information

Below is the link to the electronic supplementary material.

Supplementary Material 1

Supplementary Material 2

Supplementary Material 3

## Data Availability

The data that support the findings of this study are available upon reasonable request by contacting the corresponding author.
