# Supplementary material for: Phenolic-rich extruded BRS 305 sorghum-based beverage improves fecal and blood metabolites, oxidative balance and cardiometabolic markers in adults with excess body weight: a single-blind, randomized, placebo-controlled study
Source: Eur J Nutr. 2026 Jun 19;65(5):166. doi: 10.1007/s00394-026-04019-2 (PMC13282233; doi:10.1007/s00394-026-04019-2)
Supplement: Supplementary file 2 — Supplementary Material 2 [file 394_2026_4019_MOESM2_ESM.docx]

**Phenolic-rich extruded** **BRS 305 sorghum-based beverage improves fecal and blood metabolites, oxidative balance and cardiometabolic markers in adults** **with excess body weight: a single-blind, randomized, placebo-controlled study**

**European Journal of Nutrition**

Lucimar Aguiar da Silva^1^, Vinícius Parzanini Brilhante de São José^1^, Álvaro Luiz Miranda Piermatei^1^, Larissa Arruda Rodrigues^1^, Pietra Vidal Cardoso do Prado^1^, Renata Celi Lopes Toledo^1^, Carlos Wanderlei Piler de Carvalho^2^, Valéria Aparecida Vieira Queiroz^3^, Bárbara Pereira da Silva^1^, Joseph Francis Pierre^4^, Hércia Stampini Duarte Martino^1^

^1^ Federal University of Viçosa, (Department of Nutrition and Health), Viçosa, (Minas Gerais), Brazil

^2^ Embrapa Food Technology, Rio de Janeiro, (Rio de Janeiro), Brazil

^3^ Embrapa Maize and Sorghum, Sete Lagoas, (Minas Gerais), Brazil

^4^ University of Wisconsin-Madison, (Department of Nutritional Sciences), Madison, (Wisconsin), United States

**Corresponding author:** Hércia Stampini Duarte Martino ([hercia@ufv.br](mailto:hercia@ufv.br))

**Supplementary Material 2** Chromatograms of the sorghum and control beverages**.** Chromatographic profiles of sorghum (**A**) and control (**B**) beverages obtained by UHPLC. The peaks correspond to the twelve phenolic compounds identified in the beverages: Protocatechuic acid (**1**); *trans*-Caffeic acid (**2**); *trans*-Ferulic acid (**3**); 5-Caffeoylquinic acid (**4**); Naringenin (**5**); Quercetin (**6**); Daidzein (**7**); Eriodictyol (**8)**; Taxifolin (**9**); (+)-Catechin (**10**); Catechin (isomer) (**11**); Procyanidin B-type dimer (**12**).
