# Supplementary material for: Phenolic-rich extruded BRS 305 sorghum-based beverage improves fecal and blood metabolites, oxidative balance and cardiometabolic markers in adults with excess body weight: a single-blind, randomized, placebo-controlled study
Source: Eur J Nutr. 2026 Jun 19;65(5):166. doi: 10.1007/s00394-026-04019-2 (PMC13282233; doi:10.1007/s00394-026-04019-2)
Supplement: Supplementary file 3 — Supplementary Material 3 [file 394_2026_4019_MOESM3_ESM.docx]

**Phenolic-rich extruded** **BRS 305 sorghum-based beverage improves fecal and blood metabolites, oxidative balance and cardiometabolic markers in adults** **with excess body weight: a single-blind, randomized, placebo-controlled study**

**European Journal of Nutrition**

Lucimar Aguiar da Silva^1^, Vinícius Parzanini Brilhante de São José^1^, Álvaro Luiz Miranda Piermatei^1^, Larissa Arruda Rodrigues^1^, Pietra Vidal Cardoso do Prado^1^, Renata Celi Lopes Toledo^1^, Carlos Wanderlei Piler de Carvalho^2^, Valéria Aparecida Vieira Queiroz^3^, Bárbara Pereira da Silva^1^, Joseph Francis Pierre^4^, Hércia Stampini Duarte Martino^1^

^1^ Federal University of Viçosa, (Department of Nutrition and Health), Viçosa, (Minas Gerais), Brazil

^2^ Embrapa Food Technology, Rio de Janeiro, (Rio de Janeiro), Brazil

^3^ Embrapa Maize and Sorghum, Sete Lagoas, (Minas Gerais), Brazil

^4^ University of Wisconsin-Madison, (Department of Nutritional Sciences), Madison, (Wisconsin), United States

**Corresponding author:** Hércia Stampini Duarte Martino ([hercia@ufv.br](mailto:hercia@ufv.br))

| **Supplementary Material 3** Hypocaloric dietary plans were prescribed at the beginning of the study (restriction of 500 kcal/day) | | | | | | | | | | |
| --- | --- | --- | --- | --- | --- | --- | --- | --- | --- | --- |
| **ID** | **Group** | **Estimated Energy Requirement (EER)** | **EER - 500 kcal** | **Dietary Prescription (kcal)** | **Energy Restriction (kcal)** | **Dietary Fiber Recommendation (g)** | **Dietary Fiber Prescription (g)** | **%Carb** | **%Prot** | **%Fat** |
| 1 | Sorghum | 2030 | 1530 | 1520 | 510 | 21 | 22 | 48 | 21 | 31 |
| 2 | Sorghum | 2604 | 2104 | 2098 | 506 | 29 | 29 | 51 | 18 | 31 |
| 3 | Sorghum | 1819 | 1319 | 1313 | 506 | 18 | 19 | 51 | 19 | 30 |
| 4 | Sorghum | 1937 | 1437 | 1429 | 507 | 20 | 21 | 50 | 19 | 31 |
| 5 | Sorghum | 1919 | 1419 | 1413 | 506 | 20 | 20 | 50 | 20 | 30 |
| 6 | Sorghum | 1797 | 1297 | 1294 | 503 | 18 | 18 | 51 | 21 | 28 |
| 7 | Sorghum | 1905 | 1405 | 1401 | 504 | 20 | 20 | 50 | 20 | 30 |
| 8 | Sorghum | 1840 | 1340 | 1337 | 503 | 19 | 20 | 51 | 20 | 28 |
| 9 | Sorghum | 2101 | 1601 | 1594 | 507 | 22 | 23 | 50 | 19 | 30 |
| 10 | Sorghum | 2546 | 2046 | 2043 | 504 | 29 | 29 | 50 | 20 | 29 |
| 11 | Sorghum | 1706 | 1206 | 1204 | 503 | 17 | 18 | 51 | 21 | 28 |
| 12 | Sorghum | 1786 | 1286 | 1280 | 506 | 18 | 19 | 51 | 21 | 28 |
| 13 | Sorghum | 1978 | 1478 | 1465 | 514 | 21 | 21 | 50 | 20 | 30 |
| 14 | Sorghum | 1966 | 1466 | 1460 | 506 | 20 | 21 | 49 | 21 | 30 |
| 15 | Sorghum | 2516 | 2016 | 2014 | 502 | 28 | 29 | 51 | 21 | 28 |
| 16 | Sorghum | 1947 | 1447 | 1438 | 509 | 20 | 20 | 52 | 20 | 28 |
| 17 | Sorghum | 1700 | 1200 | 1200 | 500 | 17 | 18 | 51 | 20 | 28 |
| 18 | Sorghum | 1652 | 1152 | 1148 | 504 | 16 | 17 | 50 | 22 | 28 |
| 19 | Sorghum | 1899 | 1399 | 1399 | 500 | 20 | 20 | 50 | 20 | 30 |
| 20 | Sorghum | 1896 | 1396 | 1395 | 501 | 20 | 20 | 51 | 21 | 28 |
| 21 | Sorghum | 1880 | 1380 | 1379 | 501 | 19 | 20 | 51 | 20 | 28 |
| 22 | Sorghum | 1942 | 1442 | 1436 | 507 | 20 | 20 | 51 | 21 | 28 |
| 23 | Sorghum | 2825 | 2325 | 2319 | 506 | 32 | 33 | 51 | 21 | 29 |
| 24 | Sorghum | 2491 | 1991 | 1990 | 501 | 28 | 28 | 51 | 20 | 28 |
| 25 | Sorghum | 1783 | 1283 | 1281 | 502 | 18 | 18 | 52 | 20 | 29 |
|  |  |  |  |  |  |  |  |  |  |  |
| **ID** | **Group** | **Estimated Energy Requirement (EER)** | **EER - 500 kcal** | **Dietary Prescription (kcal)** | **Energy Restriction (kcal)** | **Dietary Fiber Recommendation (g)** | **Dietary Fiber Prescription (g)** | **%Carb** | **%Prot** | **%Fat** |
| 1 | Control | 2053 | 1553 | 1543 | 511 | 22 | 22 | 50 | 20 | 30 |
| 2 | Control | 1785 | 1285 | 1277 | 508 | 18 | 18 | 50 | 20 | 30 |
| 3 | Control | 1882 | 1382 | 1378 | 504 | 19 | 20 | 51 | 18 | 31 |
| 4 | Control | 1782 | 1282 | 1279 | 502 | 18 | 19 | 51 | 18 | 31 |
| 5 | Control | 2500 | 2000 | 1998 | 502 | 28 | 29 | 51 | 20 | 29 |
| 6 | Control | 2022 | 1522 | 1515 | 506 | 21 | 21 | 51 | 20 | 29 |
| 7 | Control | 2101 | 1601 | 1598 | 503 | 22 | 23 | 51 | 21 | 28 |
| 8 | Control | 2396 | 1896 | 1890 | 507 | 26 | 27 | 50 | 20 | 30 |
| 9 | Control | 1816 | 1316 | 1314 | 501 | 18 | 19 | 52 | 19 | 29 |
| 10 | Control | 1663 | 1163 | 1155 | 508 | 16 | 17 | 52 | 18 | 30 |
| 11 | Control | 1699 | 1199 | 1191 | 508 | 17 | 17 | 50 | 22 | 28 |
| 12 | Control | 2276 | 1776 | 1767 | 509 | 25 | 25 | 51 | 21 | 28 |
| 13 | Control | 1759 | 1259 | 1252 | 507 | 18 | 18 | 52 | 18 | 30 |
| 14 | Control | 1843 | 1343 | 1340 | 503 | 19 | 20 | 51 | 21 | 28 |
| 15 | Control | 2006 | 1506 | 1499 | 506 | 21 | 22 | 51 | 21 | 28 |
| 16 | Control | 1884 | 1384 | 1383 | 500 | 19 | 20 | 51 | 21 | 28 |
| 17 | Control | 1795 | 1295 | 1293 | 503 | 18 | 19 | 51 | 21 | 28 |
| 18 | Control | 2109 | 1609 | 1602 | 507 | 22 | 23 | 51 | 21 | 28 |
| 19 | Control | 1743 | 1243 | 1234 | 509 | 17 | 18 | 51 | 20 | 29 |
| 20 | Control | 2133 | 1633 | 1626 | 507 | 23 | 23 | 51 | 21 | 29 |
| 21 | Control | 1834 | 1334 | 1331 | 504 | 19 | 19 | 52 | 20 | 29 |
| 22 | Control | 1918 | 1418 | 1417 | 501 | 20 | 21 | 51 | 20 | 29 |
| 23 | Control | 2022 | 1522 | 1520 | 502 | 21 | 21 | 52 | 21 | 28 |
| 24 | Control | 1790 | 1290 | 1288 | 502 | 18 | 19 | 52 | 20 | 28 |
| 25 | Control | 2278 | 1778 | 1770 | 508 | 25 | 25 | 52 | 20 | 29 |
| 26 | Control | 1789 | 1289 | 1282 | 506 | 18 | 18 | 52 | 20 | 28 |
